# Implementation of a Pharmacist-Led Transitions of Care Program within a Primary Care Practice: A Two-Phase Pilot Study

**DOI:** 10.3390/pharmacy8010004

**Published:** 2020-01-04

**Authors:** Erin Slazak, Amy Shaver, Collin M. Clark, Courtney Cardinal, Merin Panthapattu, William A. Prescott, Samantha Will, David M. Jacobs

**Affiliations:** 1 Department of Pharmacy Practice, School of Pharmacy and Pharmaceutical Sciences, University at Buffalo, Buffalo, NY 14214, USA; collincl@buffalo.edu (C.M.C.); merinpan@buffalo.edu (M.P.);; 2 Department of Epidemiology and Environment Health, School of Public Health and Health Professions, University at Buffalo, Buffalo, NY 14214, USA; amyshave@buffalo.edu; 3 General Physicians, P.C., Buffalo, NY 14214, USA; ccardinal@gppconline.com (C.C.); swill@gppconline.com (S.W.)

**Keywords:** transitions of care, primary care, readmissions, pilot study

## Abstract

Pharmacists in primary care settings have unique opportunities to address the causes of ineffective care transitions. The objective of this study is to describe the implementation of a multifaceted pharmacist transitions of care (TOC) intervention integrated into a primary care practice and evaluate the effectiveness of the program. This was a two-phase pilot study describing the development, testing, and evaluation of the TOC program. In Phase 1, the TOC intervention was implemented in a general patient population, while Phase 2 focused the intervention on high-risk patients. The two pilot phases were compared to each other (Phase 1 vs. Phase 2) and to a historical control group of patients who received usual care prior to the intervention (Phase 1 and Phase 2 vs. control). The study included 138 patients in the intervention group (Phase 1: 101 and Phase 2: 37) and 118 controls. At baseline, controls had a significantly lower LACE index, shorter length of stay, and a lower number of medications at discharge, indicating less medical complexity. A total of 344 recommendations were provided over both phases, approximately 80% of which were accepted. In adjusted models, there were no significant differences in 30-day all-cause readmissions between Phase 2 and controls (aOR 0.78; 95% CI 0.21–2.89; *p* = 0.71) or Phase 1 (aOR 0.99; 95% CI 0.30–3.37; *p* = 0.99). This study successfully implemented a pharmacist-led TOC intervention within a primary care setting using a two-phase pilot design. More robust studies are needed in order to identify TOC interventions that reduce healthcare utilization in a cost-effective manner.

## 1. Introduction

Transitions of care (TOC) refers to the movement of patients between healthcare practitioners, settings, and home [1]. Ineffective transitions are common and can lead to adverse events, hospital readmissions, and higher healthcare costs [1]. Readmission rates are reported to be nearly 20% among Medicare beneficiaries, with costs of readmission estimated at $17.4 billion [2]. With the implementation of the Affordable Care Act, the Hospital Readmissions Reduction Program (HRRP) was established in 2012 [3,4]. The HRRP aimed to reduce hospital readmissions for several discharge diagnosis categories in an effort to reduce healthcare costs by financially penalizing hospitals with excessive readmissions. Such high-risk diagnoses included acute myocardial infarction, heart failure, and acute exacerbation of chronic obstructive pulmonary disease [3].

Up to 70% of patients have at least one medication discrepancy at discharge and these patients are twice as likely to be readmitted within 30 days [5,6,7,8,9]. Medication discrepancies may be attributed to patient and caregiver error or to system error because of a lack of continuity across care settings. Discrepancies are also more likely to occur as the total number of medications increases [9,10,11]. These discrepancies can cause adverse drug reactions, drug-drug interactions, and other medication-related problems that can lead to potentially preventable readmissions [12].

There has been a strong focus on facilitating safe and effective care transitions to reduce readmissions, which has led to the development of TOC programs at many institutions. Pharmacists are well-positioned to work with patients during care transitions. A recent meta-analysis demonstrated that pharmacy-led medication reconciliation interventions were an effective strategy to reduce medication discrepancies [13]. However, a separate systematic review on the role of pharmacists in care transitions found that medication reconciliation alone is insufficient for reducing post-discharge adverse clinical outcomes (e.g., readmissions, emergency department (ED) visits, and adverse drug events) [14]. The review emphasized that medication reconciliation should be combined with active patient counseling, clinical medication review, close collaboration with providers, and securing continuity of care by integrating pharmacists into multidisciplinary programs across healthcare settings.

To date, most studies describing pharmacist involvement in TOC interventions have focused on acute care settings [14]. These interventions typically involve medication reconciliation and patient education at the point of hospital discharge. There are limited data evaluating pharmacist-driven TOC interventions within primary care settings. Several early studies suggested that pharmacist involvement in primary care TOC interventions can reduce healthcare utilization following the discharge [3,15]. Hawes et al. conducted a randomized pilot study evaluating the effect of a pharmacy clinical visit after discharge and found patients randomized to the intervention had significantly lower 30-day readmission rates and ED visits [3]. Pharmacists in primary care settings have unique opportunities to address the causes of ineffective care transitions such as medication-related problems which are a major contributor to adverse clinical outcomes following discharge [16]. Additionally, pharmacists in the primary care setting can provide patient education and communicate with both patients and providers. Although primary care practices are working to incorporate these services, there is little guidance on the implementation and processes associated with a pharmacist-led primary care TOC intervention. Therefore, the objectives of this study were to describe the implementation of a multifaceted pharmacist TOC intervention integrated into a primary care practice and evaluate the effectiveness of the program.

## 2. Program Implementation and Evaluation

### 2.1. Setting

This study was conducted in a single primary care practice within one of Western New York’s largest medical groups. The medical group is a provider of primary and specialty care services throughout the greater Buffalo, NY region and includes 24 primary care clinics. The group sees on average 20,000 patients per month within their primary care clinics and approximately 52,000 patients per month across all practices. All practices within the group utilize a patient-centered medical home model that proactively manages all medical, physical, social, and behavioral healthcare needs. All clinics have a comprehensive electronic health record (EHR) system that securely tracks medical care and links with the regional health information exchange. The practice group has an established clinical pharmacy service including three full-time clinical pharmacists, one pharmacy intern, one university pharmacy faculty member, a PGY2 ambulatory care pharmacy resident, and typically, one pharmacy student completing their advanced pharmacy practice experience (APPE) rotation. The clinical pharmacy team, implemented in February 2017, supports all of the primary care offices remotely and has a physical presence in seven of the offices. In addition to transitions of care, pharmacists provide comprehensive medication management for patients with chronic disease states, patient and provider education, on-demand patient-specific drug information responses, and initiatives focused on improving medication-related quality metrics.

### 2.2. Phase 1–Implementation of Pharmacist TOC Services and Continuous Quality Improvement

Prior to the implementation of first phase of the TOC pilot program, the medical group had no standardized hospital follow-up process. Timing and location of follow-up were left to the discretion of the discharge team, with a follow-up appointment frequently advised but not scheduled. In February 2018, a TOC pilot program was implemented at a single primary care practice within the medical group (Appendix A). A registered nurse contacted the patient or caregiver by telephone and completed an initial medication reconciliation within 24 h of discharge notification being received by the primary care office. The nurse additionally scheduled a transitional care follow-up visit with the primary care practitioner (PCP). Patients with at least one medication discrepancy on initial medication reconciliation were referred to the clinical pharmacy service for a pharmacy TOC consult. Patients were eligible if they were discharged from a hospital, skilled nursing facility, or rehabilitation facility to home. The pharmacist followed up with the patient or caregiver and completed a multifaceted transition intervention. This represented a complex intervention with five distinct components tailored toward individual patient needs:

1. Review the participant’s medication records at the practice and on the discharge summary and reconcile any differences;
2. Perform a comprehensive review to identify medication-related problems, assess medication adherence, and discuss any medication changes made during the hospital admission;
3. Provide recommendations to the PCP and discuss future management plans;
4. Follow-up with the participant (if needed) and counsel on any changes made to the medication regimen;
5. Finalize updates within the participant’s EHR.

The encounter occurred either by telephone or a face-to-face appointment if the patient was scheduled to see their PCP in the office on a day the pharmacist was present. If the patient or caregiver was unavailable, the medication review was based on a thorough chart review. Any interventions accepted by the PCP were implemented immediately by the pharmacist or by the practitioner at the care transitions office appointment.

The first phase of the TOC pilot program ran for approximately four months (February 2018–May 2018) and it soon became apparent that sustainability was an emerging concern. Requests for pharmacy TOC consult services were outpacing clinical pharmacist manpower. The nature of the requests were irregular in terms of both volume and timing, and no pharmacist time was initially delegated explicitly for TOC consultations. Ultimately, fitting complex TOC consults into an already busy daily workflow became a challenge. In addition, only one PCP office was at that time included in the program, and the medical group was home to >20 primary care offices. For this intervention to be expanded and sustainable, the processes needed to be streamlined.

### 2.3. Phase 2–Process Refinement: Intervention Focus on High-Risk Patients

For Phase 2 of the TOC pilot program (October 2018–May 2019), changes were made to the program based on Phase 1 pilot data and continuous feedback from the clinical team and administration. Most importantly, eligibility criteria for a pharmacy TOC consult were updated to better identify patients at “high risk” for hospital readmission rather than the general population (Appendix A). These criteria were developed after review of Phase 1 data and implemented in October 2018. At the screening level, a registered nurse continued to perform a medication reconciliation following discharge, but patients were only eligible for a pharmacy TOC consult if they met two of the four following criteria:

(1) High-risk discharge diagnosis:
  - Cardiovascular condition (e.g., heart failure, arrhythmia, acute coronary syndrome) or cardiovascular procedure (e.g., coronary artery bypass grafting, transcatheter aortic valve replacement, stenting);
  - Acute exacerbation of chronic obstructive pulmonary disease;
  - Pneumonia;
(2) ≥15 scheduled medications upon hospital discharge;
(3) ≥2 medication discrepancies on initial medication reconciliation;
(4) Comprehensive primary care plus (CPC+) risk level of “high” as assigned by the EHR.

Subsequently, upon implementing the streamlined eligibility criteria, a second primary care office was included in December 2018. The workload for the pharmacy team remained steady in Phase 2, but efforts are currently underway to review the updated eligibility criteria.

### 2.4. Program Evaluation

This was a quasi-experimental study to evaluate program effectiveness at each phase of implementation. Each phase of the pilot was compared to the other (Phase 1 vs. Phase 2) and to a historical control group of patients who received usual care prior to the intervention (Phase 1 and Phase 2 vs. control). The study was conducted in accordance with the Declaration of Helsinki, and the protocol was approved by the Ethics Committee of the University at Buffalo (IRB#00003236).

Intervention patients included all primary care eligible patients discharged after February 2018 who had a pharmacy TOC intervention during Phases 1 or 2. Usual care patients were selected as a historical control group by reviewing hospital discharges for the same primary care office from October–December 2017, a time period predating implementation of the pharmacy TOC intervention. Patients were excluded if they were discharged to palliative care services or a long-term care facility. Patients with multiple hospital discharges during the study period had only their first admission and consultation included in the analysis.

Demographic and clinical data were abstracted from inpatient and outpatient encounters in the EHR. Data collected included demographic characteristics (age, gender, self-reported race/ethnicity, insurance status, and Charlson comorbidity index [17]). Clinical information included primary discharge diagnoses, number of medications at discharge, length of hospital stay, CPC+ risk level, and LACE score [18]. The LACE index is a tool developed to predict the risk of readmission (high, moderate, or low risk) within 30 days of discharge. It incorporates the length of stay, the acuity of the admission, patient comorbidities, and emergency department utilization in the 6 months prior to the admission. Process measures collected included the type of intervention delivery (telephonic vs. face-to-face vs. chart review only), number of days from discharge to pharmacist intervention, pharmacist recommendations and provider acceptance rates, and pharmacist time spent on TOC interventions.

To assess the effect of the intervention, first, all-cause 30-day hospital readmissions were compared between groups. All-cause readmission was defined as any unplanned patient admission to a hospital within 30 days after being discharged from an earlier hospital stay. Next, clinically-related 30-day readmissions were evaluated. To do this, the discharge diagnosis of the index hospitalization was compared with the readmission diagnoses.

Demographic and clinical data in the intervention groups (Phase 1 and 2) and control group were compared using the chi-squared test for categorical variables. A Student’s t-test or Wilcoxon rank sum test were used for continuous variables, as appropriate. The primary outcomes of all-cause and clinically-related 30-day readmissions were compared between groups with multivariable logistic regression models. Odds ratios (ORs) and 95% confidence intervals (CIs) were estimated, and each model was adjusted for predetermined confounders identified from the literature. A *p*-value < 0.05 was considered statistically significant, and all analyses were two-sided and performed using SAS v. 9.4 (SAS Institute, Cary, NC, USA).

## 3. Clinical Results and Process Measures

### 3.1. Study Sample

Patient characteristics categorized by group designation (control, Phase 1, and Phase 2) are outlined in Table 1. The population consisted of persons who were approximately 70 years of age. The majority of patients were female (control: 62.7%, Phase 1: 61.4%, Phase 2: 51.4%) and white (control: 63.6%, Phase 1: 52.5%, Phase 2: 78.4%). Most patients in the population were covered by Medicare, with nearly one-third of patients covered by commercial insurance and the remaining approximately 10% of patients covered by Medicaid. Over 50% of patients had four or more comorbidities, regardless of group. By design, a greater percentage of patients in Phase 2 had a high LACE index (70.3%) than those in Phase 1 (59.4%), whereas a majority of the control group had a moderate LACE index (66.9%). Of those receiving an intervention, most patients received it telephonically (Phase 1: 58.4%, Phase 2: 62.2%).

### 3.2. Pharmacist TOC Interventions and Acceptance Rates

A total of 344 recommendations were provided over both phases, of which 273 (79.4%) were accepted. There was no significant difference in intervention acceptance between Phase 1 and Phase 2 (78.6% vs. 81.5%; *p* = 0.55) [Table 2]. All patients in both phases were counseled on either their medication or adherence to their medication as well as having improvements to medication access completed. The pharmacist recommended an optimization of therapy in nearly 80% of patients in both phases (79.7% and 78.9%). The intervention was for an initiation of medication more frequently in Phase 1 than in Phase 2 (77.4% vs. 57.1%; *p* = 0.16). A recommendation for monitoring was made in almost 90% of the cases during both phases (87.7% and 87.5%; *p* = 0.97).

### 3.3. Percentage of 30-Day Hospital Readmissions

Compared to the control group, both Phase 1 and Phase 2 had a higher rate of 30-day all-cause readmission, although this was not significantly different (Phase 1: 16.8% and Phase 2: 13.5% vs. 9.3%; *p* = 0.09 and *p* = 0.46, respectively) (Table 3). Both phases also saw a higher percentage of clinically related 30-day readmissions compared to the control group. When comparing the two phases of the intervention, there were no significant differences in 30-day all-cause or clinically related readmissions.

### 3.4. Odds Ratios of 30-day Hospital Readmissions between Intervention (Phase 1 & 2) and Usual Care Groups

After adjusting for age, gender, LACE index, and number of medications at discharge, subjects within Phase 2 of the TOC intervention were 22% less likely to be readmitted to the hospital within 30 days for any cause compared to the control group, although this was not statistically significant (adjusted odds ratio [aOR] 0.78; 95% CI 0.21–2.89; *p* = 0.71) (Table 4). Participants in Phase 2 were just as likely to be readmitted to the hospital within 30 days for any reason compared to Phase 1 (aOR 0.99; 95% CI 0.30–3.37; *p* = 0.99). After adjusting for confounders, there was no significant relationship between Phase 2 subjects and clinically related hospital readmissions compared to the control group (aOR 4.11; 95% CI 0.52–32.3; *p* = 0.18) [Table 4]. A similar finding was seen between Phase 1 and Phase 2 subjects and clinically related readmissions.

## 4. Discussion

In this multi-phase pilot study a multidisciplinary, pharmacist-led transitions service within a primary care practice was implemented and refined. The intervention’s implementation and continued success is based on strong support from the medical group’s administration and physicians who are advocates for clinical pharmacy services. Key components of this intervention included a multidisciplinary approach, direct access to comprehensive medical records, established patient relationships, and ongoing collaborations with primary care providers. Moving forward, the plan is to further focus the service on patients who will derive the greatest benefit from pharmacy intervention and prior to expanding this service to additional primary care offices within the medical group.

Nearly 80% of pharmacist recommendations and interventions were accepted across both phases of the intervention. The high rate of recommendation acceptance in the present study is viewed as a strength and supports the value of an integrated pharmacy service within the primary care setting. The literature does not commonly report provider acceptance rates of pharmacist recommendations. It is important to note that this study included only one PCP in phase 1 and one additional PCP in phase 2, both of which are accustomed to working with pharmacists as part of the health care team. Therefore, the results may have limited generalizability. Fennelly et al. implemented a TOC comprehensive medication review intervention and had approximately 50% of their 509 recommendations accepted by the provider and over 90% accepted by the patient [19]. Intervention acceptability and fidelity are important implementation outcomes that need to be collected and reported [20]. This will be a significant next step for pharmacist-based TOC interventions in order to produce effective and lasting changes within the healthcare system.

While this study did not show a statistically significant difference in 30-day readmission rates between the intervention and control groups, this may be a result of a substantially less medically complex cohort of patients in the control group. The majority of the control group (67%) had a moderate risk of readmission based on LACE index while the majority of the phase 1 and phase 2 intervention groups had a high risk (59% and 70%, respectively). There was a trend toward lower hospital readmissions by Phase 2, although this did not reach statistical significance. Previous studies of clinical pharmacist TOC interventions in primary care have reported mixed results related to their impact on readmission rates. Hawes et al. evaluated a multidisciplinary outpatient-based transitions program focusing on moderate- to high-risk patients and demonstrated a significant increase in access to primary care and reductions in hospitalizations and ED visits [21]. Conversely, a number of studies have shown no difference in hospital readmissions after the introduction of a pharmacist-led or multidisciplinary TOC program [19,22,23]. Unfortunately, most studies in this area suffer from similar limitations such as small sample sizes and observational, retrospective designs [15,19,24,25]. Moving forward, larger studies with more robust designs (e.g., pragmatic randomized trials) are needed to identify successful interventions that reduce healthcare utilization in a cost-effective manner.

From Phase 1 to Phase 2, the focus of the TOC intervention shifted to “high-risk” patients rather than the general population. These findings indicate that a pharmacist transitions program within a general or low-risk population provides less clinical impact, in agreement with the literature [22]. The current challenge is how to identify “high-risk” patients that are more likely to benefit from clinical pharmacist interventions. An algorithm was developed to prioritize a “high-risk” population based on the pilot results, and this has successfully focused the intervention on higher risk patients. However, the volume of consults continue to increase as the intervention rolls out to additional practices. Because of sustainability concerns, the team is currently evaluating and adapting the algorithm. Recent systematic reviews comparing existing readmission predictive models highlighted that most models vary largely in their discrimination ability (C-statistics, 0.21–0.88), have low to moderate discriminative power, and lack geographic validation [26,27]. Two validated risk assessment tools commonly used in clinical practice include the HOSPITAL score and LACE index [18,28]. However, these tools are limited in that they primarily rely on variables that are unlikely to be available for use in real-time clinical practice. Therefore, there remains significant room for improvement with regard to developing risk stratification tools to assist in triaging appropriate care.

This study has several limitations. First, a relatively small number of patients were studied, limiting the ability to detect differences in outcomes between groups. However, the main goal was to describe program implementation and improvement rather than detect a difference in healthcare utilization. Next steps include conducting a larger study using a step-wedged, randomized design that evaluates this intervention and its impact on clinical outcomes. Second, only a limited number of process measures were collected, such as pharmacist recommendations and time spent on the intervention. Future studies should collect additional data on implementation outcomes such as acceptability, fidelity, and sustainability. Third, the intervention groups were poorly matched with the control group. The control group was notably less complicated, which may have led to a lower than expected readmission rate. Using a general population control group limited the ability to detect significant differences compared to Phase 1 and 2 groups. It would be prudent to prioritize higher risk groups rather than implementing these services in a broader population. Finally, as with all observational studies, there is a risk of residual confounding. While multiple confounders were adjusted for, additional factors including social information were not collected and may have impacted readmission risk. Future studies should include social characteristics when evaluating post-discharge readmission risk and TOC interventions.

## 5. Conclusions

This study describes the successful implementation of a pharmacist-led TOC intervention within a primary care setting using a two-phase pilot design. Continued evaluations are being conducted to improve the TOC intervention by focusing on high-risk patients and streamlining interventions. More robust studies are needed to demonstrate that a pharmacist-led TOC intervention embedded within primary care can reduce healthcare utilization and ultimately translate healthcare savings into a sustainable reimbursement model for pharmacists.

## Figures and Tables

**Table 1:** Baseline demographic and clinical characteristics by participation status.

Characteristic	Control n = 118	Phase 1 n = 101	Phase 2 n = 37	*p*
**Age, median (IQR)**	69 (58, 78)	68 (58, 78)	70 (61, 81)	0.67
**Gender**				0.46
Male	44 (37.3)	39 (38.6)	18 (48.7)	
Female	74 (62.7)	62 (61.4)	19 (51.4)	
**Race**				0.07
White	75 (63.6)	53 (52.5)	29 (78.4)	
Black	40 (33.9)	46 (45.5)	8 (21.6)	
Other	3 (2.5)	2 (1.9)	0	
**Prescription insurance**				0.96
Medicare	69 (58.5)	61 (60.4)	21 (56.8)	
Commercial	37 (31.4)	28 (27.7)	11 (29.7)	
Medicaid	12 (10.2)	12 (11.9)	5 (13.5)	
**CCI score, mean (SD)**	4.2 (2.4)	4.6 (2.8)	5.3 (2.8)	0.10
**median (IQR)**	4 (3, 6)	4 (3, 6)	5 (4, 7)	
**No. medications at discharge, median (IQR)**	10 (7, 14)	11 (8, 17)	11 (7, 16)	0.04 *
**Discharged from**				0.14
Hospital	113 (95.8)	90 (89.1)	33 (89.2)	
Rehabilitation facility	5 (4.2)	11 (10.9)	4 (10.8)	
**Length of stay (days), Median (IQR)**	2 (1, 4)	3 (2, 5)	3 (2, 4)	<0.001 ^^^
**No. ED visits during previous 6 months, median (IQR)**	0 (0, 1)	0 (0, 1)	0 (0, 1)	0.15
**Discharge diagnosis**				0.05
CCVD or related procedure	18 (15.3)	22 (21.8)	14 (37.8)	
COPD	2 (1.7)	4 (3.9)	0	
Pneumonia	3 (2.5)	5 (4.9)	2 (5.4)	
Other	95 (80.5)	70 (69.3)	21 (56.8)	
**LACE Index**				<0.0001 ^#^
Low	9 (7.6)	3 (2.9)	2 (5.4)	
Moderate	79 (66.9)	38 (37.6)	9 (24.3)	
High	30 (25.4)	60 (59.4)	26 (70.3)	
**Intervention delivery**				0.11
Face-to-face	-	3 (2.9)	4 (10.8)	
Telephonic	-	59 (58.4)	23 (62.2)	
No encounter	-	30 (38.6)	10 (27.0)	
**Pharmacist intervention time, minutes, mean (SD)**	-	-	33 (9.5)	
**Days from discharge to intervention, median (IQR)**	-	3 (2, 5)	6 (4, 8)	<0.0001

Abbreviations: SD, standard deviation; CCI, Charlson comorbidity index; No., number; COPD, chronic obstructive pulmonary disease; CCVD, cardiovascular and/or cerebrovascular disease; ED, emergency department; LACE, length of stay, acuity of visit, comorbidity, emergency department visit; med rec, medication reconciliation; IQR, interquartile range. Data presented as frequency (percent) or median (IQR) as appropriate; *p* represents the results of the Χ^2^-test for categorical variables and t-test or Wilcoxon rank sum, as appropriate, for continuous variables. * *p*-value indicates test for difference between all three groups; between Phase 1 and control, *p* = 0.007; between Phase 1 and Phase 2, *p* = 0.73; between Phase 2 and control, *p* = 0.10. ^^^ *p*-value indicates test for difference between all three groups; between Phase 1 and control, *p* = 0.02; between Phase 1 and Phase 2, *p* = 0.96; between Phase 2 and control, *p* = 0.13. *^#^ p*-value indicates test for difference between all three groups; between Phase 1 and control, *p* < 0.0001; between Phase 1 and Phase 2, *p* = 0.31; between Phase 2 and control, *p* < 0.0001.

**Table 2:** Pharmacist interventions and acceptance rates during study phases 1 and 2.

Interventions	Phase 1 n = 252	Phase 2 n = 92	*p*
Overall acceptance rate	198 (78.6)	75 (81.5)	0.55
Accepted Interventions			
Optimize therapy	55 (79.7)	15 (78.9)	0.94
Recommend monitoring	43 (87.8)	21 (87.5)	0.97
Discontinued/hold medication	27 (62.8)	5 (71.4)	0.66
Initiate medication	24 (77.4)	8 (57.1)	0.16
Optimize dose	13 (54.2)	11 (84.6)	0.06
Counsel on medication and/or adherence	22 (100)	11 (100)	-
Improve medication access	14 (100)	4 (100)	-

Data presented as frequency (percent); *p* represents the results of a Χ^2^-test.

**Table 3:** Comparison of all-cause and clinically related 30-day hospital readmissions.

Type of Readmission	Control n = 118	Phase 1 n = 101	*p* *	Phase 2 n = 37	*p* ^	*p* ^#^
All-cause	11 (9.3)	17 (16.8)	0.09	5 (13.5)	0.46	0.63
Clinically-related	3 (2.5)	9 (8.9)	0.04	4 (10.8)	0.03	0.73

Data presented as frequency (percent). * *p* represents the results of a Χ^2^-test comparing Phase 1 to the control group. ^^^ *p* represents the results of a Χ^2^-test comparing Phase 2 to the control group. ^#^ *p* represents the results of X^2^-test comparing Phase 1 and Phase 2 groups.

**Table 4:** Unadjusted and adjusted odds ratios for all-cause and clinically related 30-day hospital readmissions between intervention (Phase 1 and 2) and usual care groups.

Groups	OR	95% CI	*p*	aOR	95% CI	*p*
**All-cause 30-day hospital readmissions**						
Phase 1 *	1.97	0.88–4.43	0.10	1.20	0.49–2.95	0.69
Phase 2 *	1.52	0.49–4.70	0.47	0.78	0.21–2.89	0.71
**Phase 2 ^^^**	0.77	0.26–2.27	0.64	0.99	0.30–3.37	0.99
**Phase 1**	Ref.	-	-	Ref.	-	-
**Clinically-related 30-day hospital readmissions**						
Phase 1 *	3.75	0.99–14.2	0.052	2.87	0.69–11.9	0.15
Phase 2 *	4.65	0.99–21.8	0.051	4.11	0.52–32.3	0.18
**Phase 2 ^^^**	1.24	0.36–4.30	0.73	1.46	0.37–5.76	0.59
**Phase 1 ^^^**	Ref.	-	-	Ref.	-	-

Abbreviations: CI, confidence interval; OR, odds ratio; aOR, adjusted odds ratio. * Represents odds in each phase compared to odds in the control group. Adjusted models controlled for age, gender, race, LACE index, and number of medications ordered at hospital discharge. ^^^ Represents the odds in Phase 2 compared with the odds in Phase 1. Adjusted models controlled for age, gender, race, LACE index, number of medications ordered at hospital discharge, and time until completion of TOC intervention.

**Figure A1:**
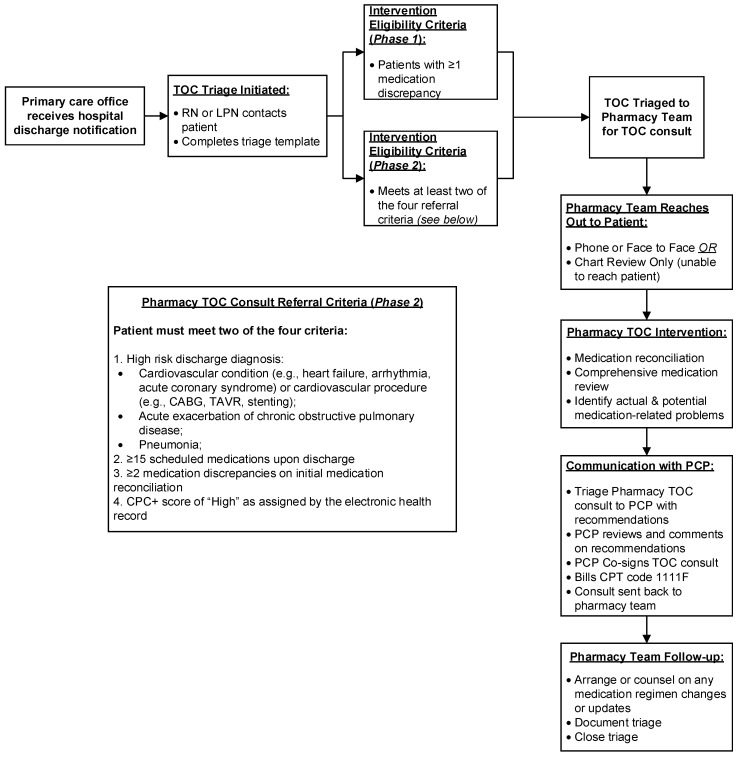
Referral Process for Pharmacy Transition of Care Consult for Phase 1 (February to May 2018 and Phase 2 (October 2018 to May 2019) (Abb. TOC, transitions of care; RN, registered nurse; LPN, licensed practical nurse; PCP, primary care provider; CPT, current procedural terminology; CPC+, comprehensive primary care plus; CABG, coronary artery bypass grafting; TAVR, transcatheter aortic valve replacement).
